# The synergistic effect of hydroalcoholic extracts of *Origanum vulgare*, *Hypericum perforatum* and their active components carvacrol and hypericin against *Staphylococcus aureus*

**DOI:** 10.4155/fsoa-2018-0096

**Published:** 2019-01-31

**Authors:** Mahmoud Bahmani, Morovat Taherikalani, Mojtaba Khaksarian, Mahmoud Rafieian-Kopaei, Behnam Ashrafi, Mohammadreza Nazer, Setareh Soroush, Naser Abbasi, Marzieh Rashidipour

**Affiliations:** 1 Razi Herbal Medicines Research Center, Department of Medicinal Plants, Lorestan University of Medical Sciences, Khorramabad, Iran; 2 Razi Herbal Medicines Research Center & Department of Microbiology, School of Medicine, Lorestan University of Medical Sciences, Khorramabad, Iran; 3 Razi Herbal Medicines Research Center & Physiology Department, School of Medicine, Lorestan University of Medical Sciences, Khorrmabad, Iran; 4 Medical Plants Research Center, Department of Pharmacology, Basic Health Sciences Institute, Shahrekord University of Medical Sciences, Shahrekord, Iran; 5 Biotechnology & Medicinal Plants Research Center, Department of Pharmacology, Ilam University of Medical Sciences, Ilam, Iran

**Keywords:** antibacterial, antibiotic, checkered carried out to Fratini method, combination, fractional inhibitory concentration, Gram positive, herbal medicine, medicinal plant, *Staphylococcus aureus*, synergist

## Abstract

**Aim:**

This study was designed to evaluate the synergistic activities of hydroalcoholic extracts of medicinal plants *Origanum vulgare* and *Hypericum perforatum* and their active components, carvacrol and hypericin against *Staphylococcus aureus*.

**Methods:**

The synergistic effects of the plants, as well as carvacrol and hypericin, were examined using a checkered method against *S. aureus* (ATCC 12600).

**Results:**

A fractional inhibitory concentration of 0.5 was obtained for combination of *O. vulgare* and *H. perforatum* and 0.49 for combination of the active ingredients carvacrol and hypericin, both of which indicated a synergistic effect.

**Conclusion:**

This preliminary evaluation demonstrated a synergistic property of *O. vulgare* and *H. perforatum* extracts in treating *S. aureus* infection. This study indicates that combination of the plants, as well as combination of carvacrol and hypericin, might be used as a new antibacterial strategy against *S. aureus*.

## Lay abstract

We studied and evaluated the synergistic activities of hydroalcoholic extracts of oregano and St John's wort and their active components, carvacrol and hypericin, against *Staphylococcus aureus*. The results suggest that a combination of oregano and St John's wort extracts, as well as a combination of hypericin and carvacrol, have potential for use as natural and effective combinations against *S. aureus* infections.

## Graphical abstract

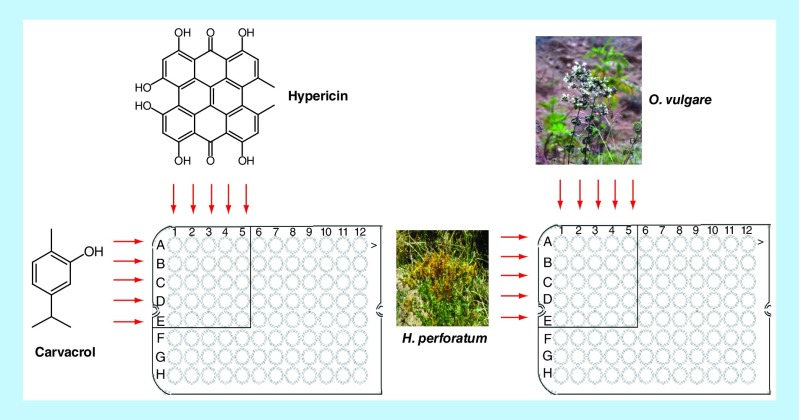

Staphylococci are Gram-positive cocci that appear in clusters. Among the different species of Staphylococci, the three species *Staphylococcus aureus*, *S. saprophyticus* and *S. epidermidis* are the most important [1]. *S. aureus* is one of the most important causes of nosocomial and community-acquired infections [2]. Due to high pathogenicity and resistance to antimicrobial and antibacterial drugs, *S. aureus* has become one of the most important health problems in the world. *S. aureus* is identified by production of coagulase enzymes and is highly pathogenic due to several extracellular toxins and factors [3]. This bacterium causes various virulent infections and food poisoning in humans [4]. It is one of the main causes of surgical wound infections in hospitalized patients and medical device-related contaminations [5]. *Staphylococcus aureus* is predominantly colonized on the surface of the skin and mucosa, and can also survive in all tissues of the body [6]. Approximately 20–40% of healthy people can be healthy carriers of *S. aureus* at any time. In some people such as hospital staff, the likelihood of being a carrier is high [7]. Approximately 30% of the population is a nasal carrier of *S. aureus*. In postoperative patients with potential *S. aureus* wound infection, microbial culture from the wound site is the most important factor for identification [8].

Both the increasing incidence of resistance to antibiotics and the side effects of these drugs have been among the factors that have led to the expansion of research on medicinal plants in recent years [9–13]. The excessive consumption of antibiotics has led to the emergence of methicillin-resistant *S. aureus* strains, which are currently one of the problems faced by hospitals [14–16]. *S. aureus* is resistant to certain types of common antibiotics, including oxacillin antibiotics (oxacillin, methysilin and colloxacillin), as well as all beta-lactam antibiotics such as penicillin, amoxicillin and cephalosporins [17]. Due to the comparatively fewer side effects of medicinal plants, their use for the treatment of various diseases has long attracted attention and has grown steadily in recent years. In the last century, the use of plant-based and natural medicinal sources as a subdiscipline of traditional medicine has played a decisive role in the prevention, control and treatment of diseases.

Considering these advantages, the tendency to use herbal drugs is increasing [18]. *Origanum vulgare* has antibacterial and antifungal effects [19,20] as well as antioxidant properties [21–24]. The main compound of *O. vulgare* is carvacrol [25,26]. *Hypericum perforatum* has antimicrobial effects [27] and hypericin is one of the most important compounds of this plant [28–31]. Plant-based antibiotics and their synergistic effects could be a useful and practical solution to prevent antibiotic resistance. Studies of synergistic effects of plant extracts are therefore necessary to identify new combinations with highly desirable efficacy. Despite the obtained valuable information about the medicinal plants *O. vulgare* and *H. perforatum*, and their active compounds such as carvacrol and hypericin, their synergistic effects have not yet been studied. The current study is a preliminary evaluation of antibacterial and synergistic activities of the extracts of medicinal plants *O. vulgare* and *H. perforatum* and their active components, carvacrol and hypericin, against *S. aureus*.

## Methods

### Preparation of hydroalcoholic extracts & active ingredients of medicinal plants

To prepare the hydroalcoholic extracts, the plants were first dried in laboratory conditions and then 200 g of the powder of each plant was mixed with ethanol 70% (Nasr Alcohol, Iran). The mixture was shaken for approximately 6 h and then left in the laboratory for 24 h. The mixture was then passed through a filter paper, and the solvent was separated from the extract using a distiller (IKA^®^ RV10) in vacuum (rotary) conditions at 40°C and 150 r.p.m. The concentrated extract of the plant was poured into the plate to dry.

### *Staphylococcus aureus* bacterium

*Staphylococcus aureus* strain (ATCC 12600) was purchased from Iranian Research Organization for Science and Technology.

### Synergism protocol

In order to investigate the combined effects of hydroalcoholic extracts of *O. vulgare* and *H. perforatum* and their active ingredients hypericin and carvacrol, the following concentrates were prepared for each of the compounds according to the amount of minimum inhibitory concentration (MIC), which was previously separately measured (4MIC_0_, 2MIC_0_, MIC_0_, MIC_0_, MIC_0_/2 and MIC_0_/4).

The combination effects of hydroalcoholic extracts of *O. vulgare* and *H. perforatum* against *S. aureus* were investigated using checkerboard test in a sterile 96-well plate. First, 50 μl of sterile Mueller–Hinton agar growth medium was added to all wells, then the plant extract samples were treated with different concentrations of the extract (20 μl of each extract). Then, 10 μl of microbial suspension with 0.5 McFarland standard turbidity (1.5 × 10^8^ CFU/ml) was added to each wall. The plates were incubated at 37°C and 50% humidity for 24 h [29].

Bacterial growth inhibition was measured by 2,3,5-triphenyltetrazolium chloride, in such a way that if the color of the wells turned purplish, the bacteria in the wells were considered living, and lack of the color was considered to indicate bacterial growth inhibition. The results were analyzed using the formula below and interpreted as follows:
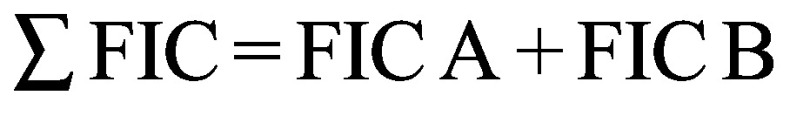



FIC A = Combination effect/MIC A: The effect of MIC A alone.

FIC B = Combination effect/MIC B: The effect of MIC B alone.

Interpretation of the obtained results of proposed model by checkered method carried out according to Fratini *et al*. was as follows: if the results are less than 1 (FIC < 1), the effect is synergistic [29]. If the results are equal to 1 (FIC = 1), then the effect is indifferent. If the results are greater than 1 (FIC > 1), this indicates an antagonistic effect, and if the results are greater than 2 (FIC > 2), one of the combination drugs is above its effective dose [29].

### Study of synergism by disc diffusion method

To investigate synergism using a disk diffusion method, the checkered method carried out according to Fratini *et al*. was used with a minor modification [29]. In brief, a sterile Mueller–Hinton agar growth medium was divided into 10-cm plates, and using a sterile swab, a grass culture was prepared from 0.5 McFarland standard turbidity obtained from 24 h culture of *S. aureus*. Then, the disks were impregnated with 40 μl of 10,000 μg/ml of stock solution of the *O. vulgare* and *H. perforatum* extracts. They were also impregnated with 40 μl of 5000 μg/ml of stock solutions of hypericin and carvacrol and placed on each other as double in the Mueller–Hinton agar growth medium. Each of the above was used as a control in a separate experiment, then the plates were incubated for 24 h, and the diameter of the growth inhibition zone was measured.

## Results

**Figure 1.**
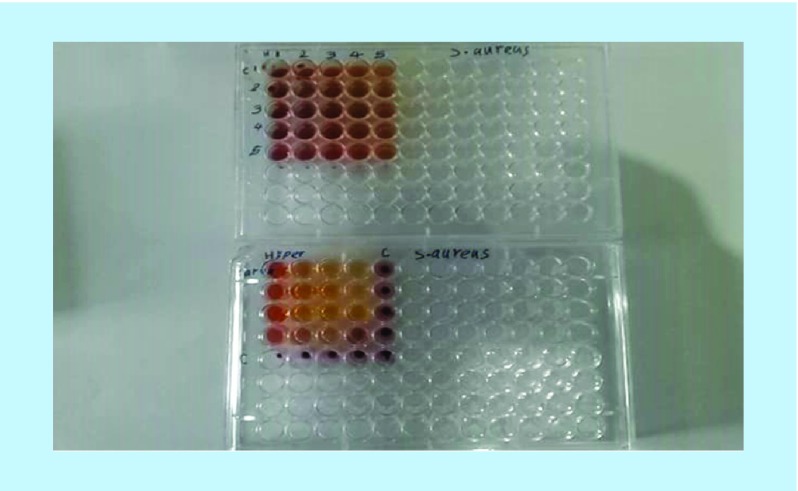
**Synergistic effects of combination of hydroalcoholic *Origanum vulgare* and *Hypericum perforatum* extracts, and active ingredients carvacrol and hypericin by means of checkerboard test in a 96-well microplate.**

**Figure 2.**
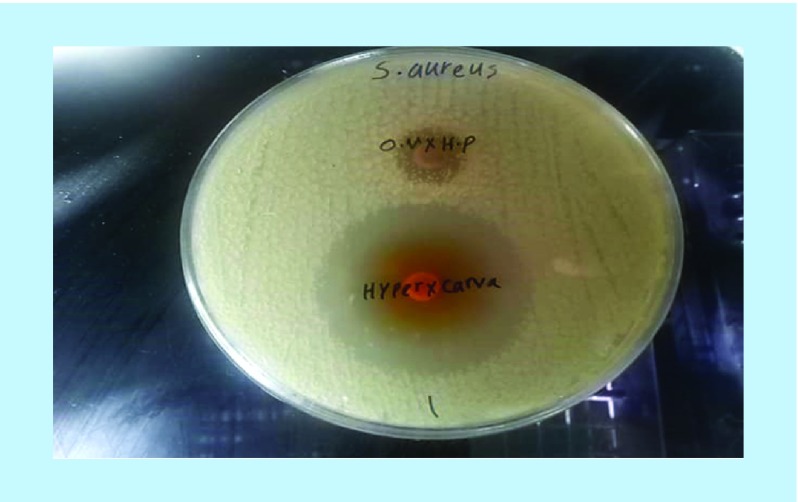
**Synergistic effect of combination of hydroalcoholic *Origanum vulgare* and *Hypericum perforatum* extracts, and active ingredients carvacrol and hypericin by means of Fratini double-antibiotic synergistic test.**

A fractional inhibitory concentration (FIC) of 0.5 was obtained for combination of hydroalcoholic extracts of *O. vulgare* and *H. perforatum* and 0.49 for combination of the active ingredients carvacrol and hypericin, indicating synergistic effects for both of them (Table 1 and Figures 1 & 2). Additional information is shown in Table 1.

**Table 1.** **Results of fractional inhibitory concentration and disk diffusion of the groups.**

**Groups**	**Minimum inhibitory concentration**	**Fractional inhibitory concentration**	**Disk diffusion**	**Ref.**
*Origanum vulgare*	625		15.66	[32]

*Hypericum perforatum*	625		12.66	

Hypericin	78.12		36	

Carvacrol	312.5		12.33

*O. vulgare* × *H. perforatum*		0.5	21

Hypericin × Carvacrol		0.49	51	

## Discussion

The results of the present study, which examined the inhibitory effects of the extracts and their active ingredients on *S. aureus*, demonstrated synergistic effects of both combinations on the studied pathogen and strengthening of the antibacterial effects on this pathogen.

Interestingly, the results on the synergistic effects in the disk diffusion test also showed a direct correlation with the inhibitory effect, so that the growth inhibition zones in the combination test for both the active ingredients and the extracts were greater than those in the combination test for either alone. In these tests, the extracts and active ingredients showed synergistic effects, probably due to the presence of common active ingredients in the plants, namely carvacrol and hypericin. This was also confirmed in the test of active ingredients and their synergistic effects.

*O. vulgare* has antimicrobial effects on Gram-positive and Gram-negative bacteria [33–37]. *H. perforatum* has a range of antimicrobial activities against bacteria, viruses, fungi and yeasts [38–42]. Unlike antibiotics and chemotherapeutic agents, few studies have so far addressed the potential mechanisms for production of plant-derived products [43]. Plant-based and natural compounds, as well as their active ingredients, may work through mediating metabolism by activating enzymes, inhibiting the function of inhibitors that affect nutrients in the environment, interfering with enzymatic processes at the nucleus or ribosome level, inducing changes in the membrane or even interfering with secondary metabolism [44]. The synergistic compounds in this study are likely to exert their effects through one or more than one of these mechanisms.

This study confirmed the synergistic properties of hydroalcoholic *O. vulgare* and *H. perforatum* extracts against *S. aureus*. Combination of hydroalcoholic *O. majorana* extract and *H. perforatum*, as well as combination of carvacrol and hypericin will allow us to use a lower concentration of the extract or their active ingredients, hence reducing the possible toxic effects.

Fratini *et al*. (2017) showed that essential oil of *O. vulgare* L. and *Leptospermum scoparium* have synergistic effects against *S. aureus* [29]. In general, herbal plants in the laminaceae family are known for their antimicrobial effects, which is due to high levels of phenol compounds such as carvacrol and thymol. It has been shown that the cardinal action mechanism of carvacrol on bacterial cells involves the decomposition of proton-motive force and the drainage of the ATP pool with blood cells [45]. The only antibacterial principle isolated to date is a hypericin, hyperforin and tetraketone [46].

In our study, the synergistic effect of carvacrol and hypericin was 0.49, and the synergistic effect of *O. vulgare* and *H. perforatum* was also 0.5. One of the reasons why the combination of carvacrol and hypericin has a better effect than *O. vulgare* and *H. perforatum* is the purity of effective compounds. Overall, *O. vulgare* and *H. perforatum* extracts and also carvacrol and hypericin may be an effective alternative to chemotherapeutic drugs in staphylococcal infections.

## Conclusion & future perspective

The present study provided evidence of the antimicrobial and synergistic effects of the combination of hydroalcoholic *O. vulgare* and *H. perforatum* extracts, as well as combination of carvacrol and hypericin, on *S. aureus* infection. This suggests that, in the future, this combination could be used as a polyherbal antibiotic compound to control bacterial infections, especially of *S. aureus*. We did not examine the possible synergistic effects of the plant extract or their active ingredients with commercial antibacterial agents. If they possess synergistic activity with commercial antibiotics, this could have several beneficial effects for patients. This synergistic activity could enable the reduction of doses of commercial antibiotics, which in turn would reduce their toxic effects. Furthermore, infection is always associated with oxidative stress. Therefore, these plants with their antioxidant properties may reduce the injuries associated with these infections.

**Executive summary**

- This experimental study was designed to evaluate the antibacterial and synergistic activities of hydroalcoholic extracts of herbs of *Origanum vulgare* and *Hypericum perforatum* and their active components, carvacrol and hypericin, against *Staphylococcus aureus*.
- The synergistic effects of *H. perforatum* and *O. vulgare*, and carvacrol and hypericin, were examined using a Checkerboard test and AZDAST test against *S. aureus*.
- A fractional inhibitory concentration of 0.5 was obtained for combination of *O. vulgare* and *H. perforatum* and 0.49 for combination of the active ingredients carvacrol and hypericin, both of which indicate a synergistic effect.
- The results of this study indicate that combination of *O. vulgare* and *H. perforatum*, as well as combination of carvacrol and hypericin, might be used as a new strategy for antibacterials against *S. aureus* strain.
